# Effects of Artificial Hydrothermal Aging on Crush Boxes Made from Glass, Carbon and Aramid Fiber-Reinforced Hybrid Composites

**DOI:** 10.3390/polym18020249

**Published:** 2026-01-16

**Authors:** Baran Erkek, Mehmet Şükrü Adin, Ertan Kosedag, Mateusz Bronis, Hamit Adin

**Affiliations:** 1 Van Vocational School, Van Yuzuncu Yil University, Van 65080, Turkey; 2 Besiri OSB Vocational School, Batman University, Batman 72060, Turkey; 3 Faculty of Engineering, Van Yuzuncu Yil University, Van 65080, Turkey; 4 Department of Machine Design and Machining, Kielce University of Technology, 25314 Kielce, Poland; 5 Faculty of Engineering and Architecture, Batman University, Batman 72100, Turkey

**Keywords:** crush boxes, crashworthiness behaviour, energy absorption, fiber type effect, graphene, hybrid composites, hydrothermal aging

## Abstract

Vehicle crush boxes are one of the safety elements used in vehicles to minimize damage that may occur during an accident. The task of crush boxes is to absorb the energy which is generated during an accident. In this study, peak force, energy absorption and specific energy absorption values of cylindrical composite crush boxes, to which 0.25% and 0.50% graphene was added, were experimentally investigated with hydrothermal aging. The composite crush boxes were produced with vacuum infusion method. Glass, aramid and carbon fibers and their hybridizations were used as fibers. During hybridization, the winding order of the fibers was changed from inside to outside. The parameters for hydrothermal aging were selected as 500 h and 1000 h at 60 °C. The highest energy absorption value was obtained in the carbon fiber-reinforced sample CFRPG1H2 with 0.25% graphene-added epoxy resin matrix, aged for 1000 h. The lowest peak strength was observed in the aramid fiber-reinforced sample AFRPG2H2 with 0.50% graphene-added epoxy resin matrix, hydrothermally aged for 1000 h. It was observed that increasing the graphene addition rate reduced the negative effects on aging. It was determined that increasing the graphene ratio by 0.25% had an effect on aging.

## 1. Introduction

In accidents, the vehicle integrity may be compromised, passengers may be injured, or even more severe outcomes may occur. Crush boxes are safety elements mounted on the chassis of vehicles to minimize possible damage in these cases. When metal materials are used, they add extra weight to the total weight of the vehicles. For this reason, recent studies have been directed towards composite materials that are at least as strong as metals, while being significantly lighter.

Many factors such as the geometry of the crush boxes, the materials used, the fiber structure, the fiber winding angle and hybridization affect the mechanical properties [1,2]. In addition to these, continuous operation under certain conditions is another factor affecting the mechanical properties of the materials. Researchers are working to obtain the best results by changing many variables such as the types of materials, their geometry and artificial aging. In hydrothermal aging, water accelerates structural changes due to increased polymer mobility in the plasticized glassy network; accordingly, chemical degradation mechanisms are affected. These changes have an effect on the performance of an adhesive joint made with this epoxy, a coating or a fiber-reinforced composite [3]. El Baky et al. [4] investigated the hydrothermal aging parameters of jute/glass/carbon hybrid composites. The samples were kept in seawater and pure water at 60 °C for 60 days. They found that the sample absorbed pure water more strongly than seawater. They also observed that non-hybrid jute composites absorbed more water than other hybrid groups. The water recovery of non-hybrid jute composite was determined as 17.44% in pure water and 16.5% in seawater. Chackraverty et al. [5] investigated the effects of hydrothermal immersion and hygrothermal conditioning on the mechanical properties of glass fiber-reinforced epoxy composite. They created an environment with 95% relative humidity at 60 °C as hydrothermal conditioning and distilled water immersion at 65 °C as hygrothermal conditioning. They found that the interlaminar shear strength values deteriorated by 23% after 120 days of hydrothermal immersion and by 25% after 90 days of hygrothermal conditioning. Soykok et al. [6] investigated how mechanically fastened connections of glass fiber/epoxy composites were affected by hot water immersion. Specimens were exposed to hot water at temperatures of 50, 70 and 90 °C for 1 and 2 weeks before the connection assembly and testing. After performing tensile tests, they concluded that the failure behavior of the connections in the aged samples was closely related to the applied temperature and the duration of hot water immersion. Mourad et al. [7] investigated the hydrothermal aging effect of seawater on glass composites. Glass/epoxy and glass/polyurethane composite samples were aged in seawater at room temperature and 65 °C for 12 months. They stated that as the aging time increased, the water uptake of both composite systems increased, and the maximum water recovery of glass/epoxy composites aged at room temperature and 65 °C was 2.5% and 5%, respectively. Malik et al. [8] investigated the effects of hydrothermal aging on basalt fiber-reinforced thermoplastic composites. They stated that the composites not subjected to aging were the best in terms of mechanical properties. Zhang et al. [9] studied the hydrothermal aging of carbon fiber-reinforced polyether ether ketone composites. They stated that the adhesion of polyether ether ketone to the carbon fiber surface weakened and mechanical properties decreased as a result of aging. Kuzmina et al. [10], in their research on the aging of basalt-reinforced composites, stated that material degradation occurred due to deterioration in fiber–matrix interface connections and that critical loads decreased.

Along with aging studies, there are studies where the structure and geometry of the material are changed and sometimes hybridization and filling are made [11,12,13,14,15,16,17,18]. Many studies have been conducted indicating that the energy absorption increases when the material is made of metal composite compared to material used in plain metal [19,20,21,22]. Zhang et al. [23] investigated the energy absorption properties of tapered circular tubes with stepped thickness under axial loading. They stated that the energy absorption efficiency of the tapered circular tubes produced with stepped thickness is quite high compared to the plain tubes, and that the shaping effects have an important effect on the efficiency increase. Yıldırım et al. [24] investigated the torsional stresses of hollow circular shafts produced from composite materials and having different orientation angles. They determined that the modulus of rupture increases as the orientation angle of the fiber increases.

Another issue that has attracted the attention of researchers is the addition of reinforcement elements to composite materials. Hegazy et al. [25] conducted a study on the lateral impact response of thin-walled composite structures filled with carbon nanopowder. The general results showed that the addition of carbon nanopowder up to 0.50 wt% increased both the energy absorption and specific energy absorption. El-Baky et al. [26] tested glass/epoxy composite tubes reinforced with five types of nanofillers, namely halloysite nanoclay (HNC), montmorillonite clay (MC), alumina (Al_2_O_3_), silica (SiO_2_) and silicon carbide (SiC). They observed that the energy absorption increased compared to pure glass epoxy. Alshahrani et al. [27] investigated the effect of the addition of halloysite clay nanotubes on the impact durability performance of glass/epoxy composite tubes. It was observed that the addition of halloysite clay nanotubes increased the energy absorption capacity of glass/epoxy composites. Alshahrani et al. [28] experimentally investigated the quasi-static crushing response and energy absorption of glass-reinforced epoxy thin-walled crush box filled with nano-aluminum oxide (Al_2_O_3_). They observed that the addition of nano-aluminum oxide had a positive effect on the energy absorption. Gupta et al. [29] investigated the effects of graphene nanoparticles on carbon fiber-reinforced composites. In their study, they used graphene addition at 0.1%, 0.2%, 0.3% and 0.4% by weight. They stated that the best mechanical properties were obtained with the addition of 0.3% graphene.

Previous studies have generally been conducted using variables such as the manufacturing method, geometry and type of material used for the crush box. The aging parameters used were performed by measuring the effects of temperature and time in an artificial aging environment. The hybridization generally stands out as metal–composite hybridization. The aim of this study is to produce composite crush boxes, traditionally manufactured by filament winding, using a different method: vacuum infusion. Furthermore, we aim to experimentally investigate the effects of hydrothermal aging on maximum peak force, energy absorption and specific energy absorption in crush boxes produced with two different graphene addition rates (0.25% and 0.50%). Glass, aramid and carbon fiber, which are widely used in the market as material structures, and crush boxes produced by hybridizing them, were subjected to quasi-static testing and compared. The aging process was performed by subjecting the material to artificial hydrothermal aging at 60 °C for 500 h and 1000 h.

## 2. Materials and Methods

### 2.1. Material Properties

**Figure 1:**
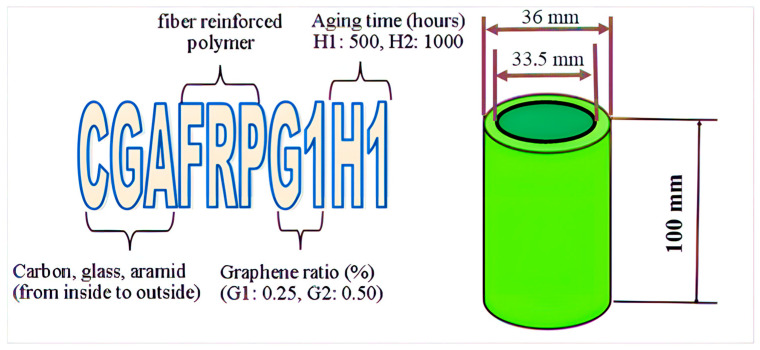
Nomenclature and geometric dimensions of a sample.

While producing composite materials, glass, aramid and carbon fibers were selected as 200 g/m^2^. The properties of these fibers are given in Table 1. Epoxy resin was used as matrix element. Hegzon LR160 and Hegzon LH160 were used as epoxy resin and hardener, respectively. The fibers, epoxy resin, hardener and other materials used for production were sourced from Dost Kimya Limited-Türkiye. The properties of epoxy are given in Table 2. Sample dimensions were produced based on the 1/3 (diameter/length) ratio in the literature [11]. The nomenclature and geometric dimensions of a sample specimen are presented in Figure 1. The inner diameter of the produced samples was 33.5 mm and, accordingly, their lengths were 100 mm. The outer diameter of the samples varies according to the fiber type because the wettability of each fiber with epoxy resin is different and this affects the thickness of the sample. Production was carried out by winding the fibers on bobbins of equal lengths. Winding was performed in a way that there would be 6 layers in total. Hybrid samples were wound in 6 layers in total, each fiber in 2 layers. During hybridization, the fibers were wrapped in a sequence by changing the winding order from inside to outside, and a total of 3 different hybridizations were made. Graphene additions were selected at 0.25% and 0.50%. In order to investigate the aging effect, the produced samples were kept in 60 °C pure water for 500 h and 1000 h. Table 3 provides information about the names of the samples, fiber structures, graphene addition rates in percentage, and aging times.

### 2.2. Preparation of Composites

**Figure 2:**
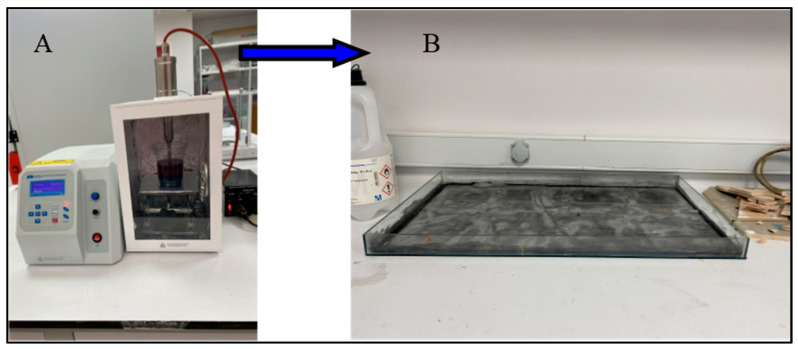
The process of incorporating graphene into fibers: (**A**) ultrasonic mixing process, (**B**) soaking the fiber in acetone–graphene solution.

**Figure 3:**
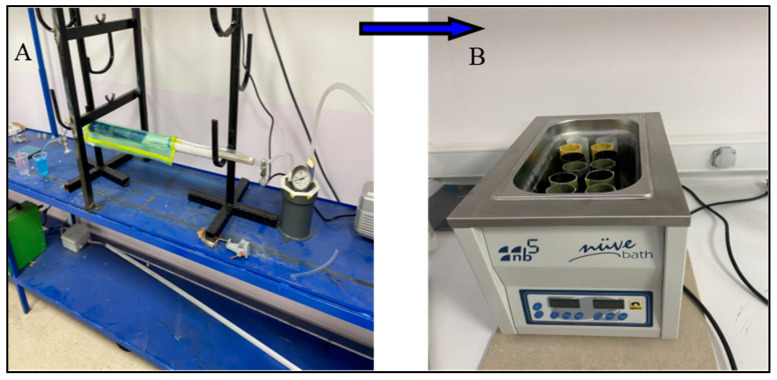
Production processes: (**A**) epoxy transfer by vacuum infusion, (**B**) hydrothermal aging process.

**Figure 4:**
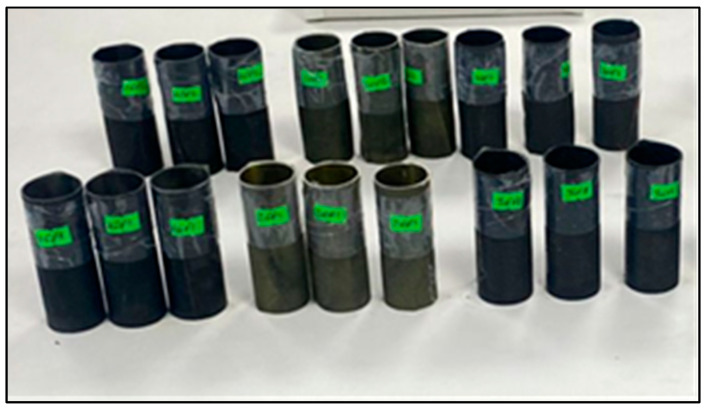
Samples ready for testing upon completion of the production and aging procedures.

Before preparing the composite crush boxes, the outer diameters of the 33.5 mm bobbins were sealed. In order to add graphene to the fibers at the specified rates, 0.25% and 0.50% graphene was dissolved in acetone solution according to the epoxy resin weight. It was mixed in an ultrasonic mixer for 10 min for a homogeneous mixture. After the mixture was finished, it was poured onto the fiber surface in a container and left to stand (Figure 2). The acetone that was left at room temperature for 24 h evaporated, leaving behind only the graphene distributed homogeneously on the fiber. The fibers that were ready for winding were wrapped on bobbins and made ready for vacuum infusion. The aim of the vacuum infusion method is to ensure that the epoxy resin given to the samples is distributed equally everywhere. The samples that are kept at room temperature for one day complete their curing and are ready for cutting. The samples cut to be 100 mm long were placed in a device called Nuve Bath for hydrothermal aging process (Figure 3). The samples were subjected to hydrothermal aging in pure water for 500 h and 1000 h and were ready for testing (Figure 4).

### 2.3. Crush Test Procedures

A quasi-static compression test was applied to find the energy absorption and maximum peak force of the samples. This process was carried out at room temperature. The test parameter was selected as 2 mm/s. The brand of the test device is RAAGEN brand ETM-50-S. A new force–displacement graph was created with the data obtained during this experiment. The area under this curve was calculated with the help of the MATLAB R 2020a package program. This calculated area gave us the energy absorption obtained by the samples. The traps command was used to calculate this area. After obtaining the energy absorption, we calculated the specific energy absorption that we used to compare different samples. In doing this, we performed the process by dividing the energy absorption of the samples by their weights. Related formulas:

(1) $$SEA=\frac{EA}{m}$$

## 3. Results and Discussion

In this section, the force–displacement graphs obtained as a result of the compression test of GFRPG1H1—GACFRPG2H2 samples were evaluated. The effect of hydrothermal aging on the maximum peak force, energy absorption and specific energy absorption of the samples reinforced with 0.25% and 0.50% graphene was compared from these curves. A comparison was made with the samples without graphene addition obtained from our previous study [1] and with 0.25% and 0.50% graphene added in another study [2]. When calculating the specific energy absorptions, which are important for comparing different types of fibers, we divided the energy absorptions by the sample weights.

### 3.1. Results for GFRPG1H1—GACFRPG1H1 Samples

**Figure 5:**
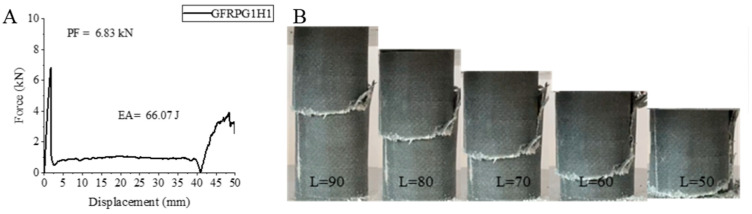
Sample GFRPG1H1: (**A**) force–displacement curve, (**B**) length changes due to deformation in quasi-static testing.

The force–displacement graph for our first sample, GFRPG1H1, and the shape changes during and after the test are given in Figure 5. Glass fiber was used as the fiber structure of the GFRPG1H1 sample and this sample with 0.25% graphene addition was subjected to 500 h of hydrothermal aging. As can be seen from Figure 5, the maximum peak force was determined as 6.83 kN. Energy absorption was calculated as 66.07 joules. When we divided this value by the sample weight (Table 4), we obtained the specific energy absorption as 2.57 J/g.

When compared with the sample with 0.25% graphene addition and not hydrothermally aged in our previous study [2], a 36% increase in maximum peak force was obtained. Increases of 203.35% and 182.41% were observed in energy absorption and specific energy absorption, respectively. These values are lower than the values obtained in our study except for PF [1] without graphene addition and without aging. The maximum peak force in this sample compared to the unaged sample without graphene addition. EA and SEA increased by 17.15%, decreased by 68.80% and 72.45% decrease was observed, respectively. Although aging negatively affects the characteristic properties of the material on the basis of aging, it slightly reduced the brittleness obtained with graphene addition in this sample and positively affected it. Matrix fracture was observed during the experiment. At the same time, the fibers also experienced fracture, but delamination was not observed. The sample was broken and intertwined.

The second sample, AFRPG1H1, is produced with aramid fiber and is a sample with 0.25% graphene added. After applying 500 h of hydrothermal aging process to this sample, the effects of aging were evident in PF, EA and SEA values compared to the sample with 0.25% graphene added. PF, EA and SEA values were determined as 0.77 kN, 39.18 joules and 3.39 j/g, respectively. And these values decreased by 47.36%, 10.07% and 17.91%, respectively, compared to the sample with 0.25% graphene added [2].

**Figure 6:**
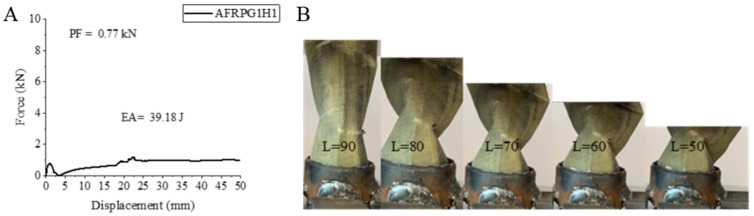
Sample AFRPG1H1: (**A**) force–displacement curve, (**B**) length changes due to deformation in quasi-static testing.

Since the aramid fiber sample is a sample with good water absorption and a sample that interacts well with graphene, it has not been aged at all and has given better results than the sample without graphene, except for SEA. Compared to the unaged sample without graphene addition [1], PF, EA and SEA values increased by 8.45%, 26.42% and 0.05%, respectively. SEA is not a value to be taken into consideration. Since good water absorption gives flexibility to this sample, a special apparatus was made during the experiment to prevent the sample from being thrown out between the jaws until it reached 50 mm during semi-static compression (Figure 6). No breakage was observed during the experiment. It showed a tendency to bend while preserving its matrix and fiber structure. Although it tended to return to its original state after the experiment, it could not reach its original size.

**Figure 7:**
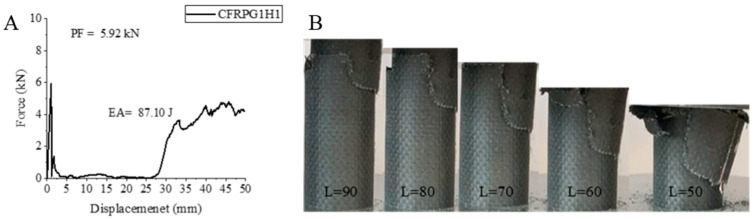
Sample CFRPG1H1: (**A**) force–displacement curve, (**B**) length changes due to deformation in quasi-static testing.

The third sample, carbon fiber sample AFRPG1H1, has thoroughly exhibited the effects of 500 h of aging. There was a 28.06% decrease in the maximum peak force with 5.92 kN. EA and SEA values were calculated as 87.1 joules and 3.54 j/g, respectively. These values are 61.84% and 62.42% lower than the sample without 0.25% graphene. The carbon fiber sample was generally negatively affected by graphene addition and aging. Its own allotrope, graphene addition, caused a decrease in its values [2]. The values were also lower than the sample that was not aged at all and without graphene. After the experiment, peeling was observed by opening the sample up like a flower (Figure 7). Matrix and fiber fractures were observed. The parts where the fracture occurred formed a pile on the material. All of these observations were in accordance with the force–displacement curve.

**Figure 8:**
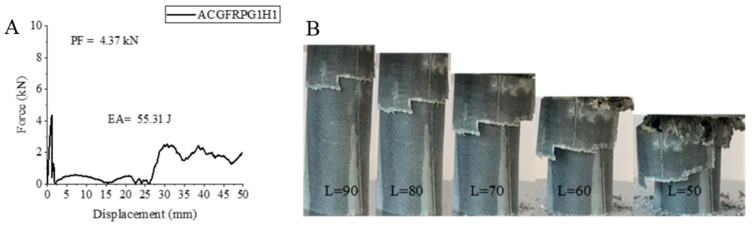
Sample ACGFRPG1H1: (**A**) force–displacement curve, (**B**) length changes due to deformation in quasi-static testing.

The force–displacement curve of the ACGFRPG1H1 sample, its change during the experiment and the final state of the sample after the experiment are given in Figure 8. This sample is one of the hybrid samples and the winding order is aramid in the innermost, carbon in the middle and glass fiber in the outermost part, 0.25% graphene added and hydrothermally aged for 500 h. Although aging did not have much effect on the maximum peak force, a decrease was observed in the EA and SEA values.

The maximum peak force increased by 0.01 kN with 4.37 kN compared to the unaged sample. The EA value decreased by 29.41% with a value of 55.31 joules compared to the sample with 0.25% graphene added and not subjected to hydrothermal aging. The situation is similar in SEA, but a decrease of 17.88% was observed with a value of 2.47 j/g. The internal peelings observed during the experiment are important for the hybrid samples. In this sample, which has fiber aramid in the innermost part, delamination was observed together with matrix and fiber fractures. However, although the aramid in the inner part tried to hold the structure without breaking, as in the S2 sample, it did not produce favorable outcomes. This sample also did not have graphene added and exhibited a decrease of 14.4%, 55.64% and 61.34% in maximum peak force, EA and SEA values, respectively, compared to the unaged sample.

**Figure 9:**
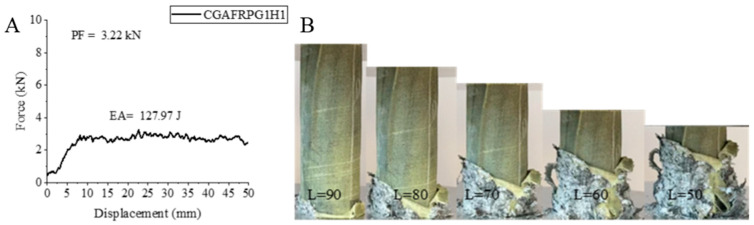
Sample CGAFRPG1H1: (**A**) force–displacement curve, (**B**) length changes due to deformation in quasi-static testing.

The other hybrid sample, CGAFRPG1H1, has the carbon fiber sample in the innermost part, where we obtained the best values individually. The shape change and force–displacement curve of this sample, which was aged 500 h hydrothermal with 0.25% graphene added, glass fiber in the middle part and aramid fiber in the outermost part, is given in Figure 9. The maximum peak force decreased by 40.14% compared to the unaged sample with 0.25% graphene added and reached the value of 3.22 kN. EA and SEA values were also affected by aging, and 127.97 joules and 6.64 j/g values were determined, respectively. These values are 7.10% and 12.63% lower than the unaged sample, respectively. Crushing was observed during the experiment. Matrix fractures occurred. At the end of the experiment, a crushed state was observed with a flower-like structure. Among the hybrid samples, this sample, which had the best EA and SEA values, showed lower results than the unaged and graphene-free sample, as in the other samples. PF, EA and SEA values decreased by 49.21%, 23.14% and 12.28%, respectively.

**Figure 10:**
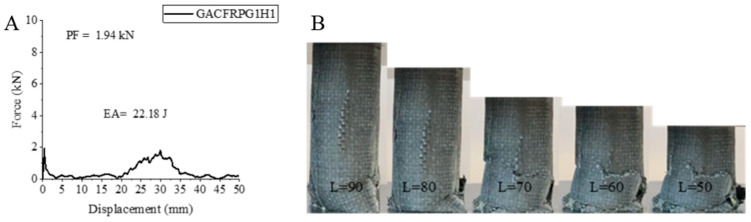
Sample GACFRPG1H1: (**A**) force–displacement curve, (**B**) length changes due to deformation in quasi-static testing.

**Figure 11:**
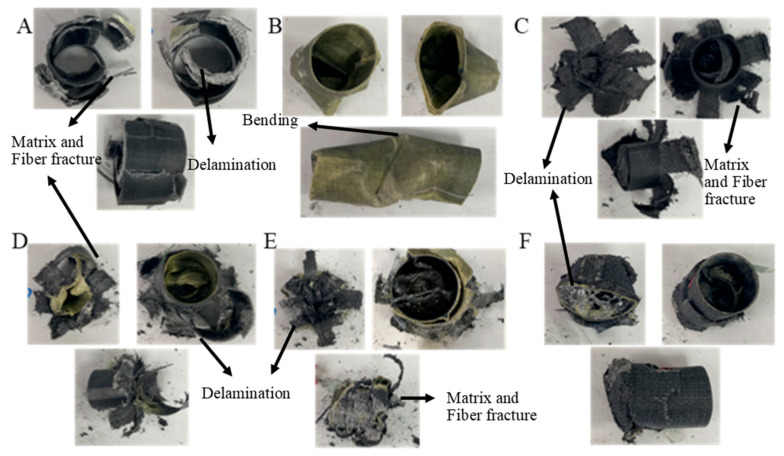
Images of samples at the end of the experiment: (**A**) GFRPG1H1, (**B**) AFRPG1H1, (**C**) CFRPG1H1, (**D**) ACGFRPG1H1, (**E**) GAFRPG1H1, (**F**) GACFRPG1H1.

In the GACFRPG1H1 sample, where the glass fiber is located in the inner part, there is aramid fiber in the middle part and carbon fiber in the outer part. This sample with 0.25% graphene added exhibited decreases in PF, EA and SEA values with 500 h of aging. PF, EA and SEA values were determined as 1.94 kN, 22.18 joules and 1.12 j/g, respectively. Compared to the unaged sample with 0.25% graphene added, PF decreased by 51.86%, EA by 56.62% and SEA by 56.58%. Matrix fractures were observed during the experiment (Figure 10). Delamination, fiber fractures and crushings were also seen in this sample (Figure 11). The presence of aramid fiber in the middle part and its flexible structure with aging demonstrated a tendency to keep the structure together. The decreases in this sample compared to the unaged sample without graphene addition are 64.72%, 81.98% and 81.78% for PF, EA and SEA, respectively.

The values for GFRPG1H1-GACFRPG1H1 samples are given in Table 4. Images of the samples at the end of the experiment are given in Figure 11.

### 3.2. Results for GFRPG1H2—GACFRPG1H2 Samples

**Figure 12:**
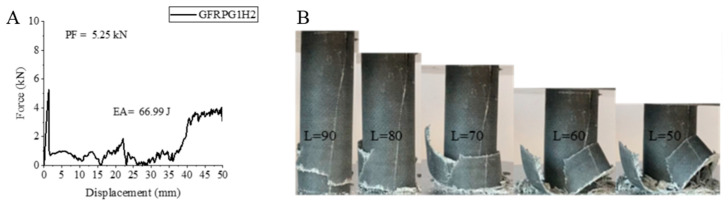
Sample GFRPG1H2 (**A**) force–displacement curve, (**B**) length changes due to deformation in quasi-static testing.

Although increasing the hydrothermal aging process from 500 h to 1000 h caused a decrease in the values, from time to time, small increases were observed. In Figure 12, the force–displacement curve of the 1000 h aged GFRPG1H2 sample with 0.25% graphene addition and its final state during and after the experiment are given. The peak force of 1000 h hydrothermal aging decreased by 23.13% compared to 500 h hydrothermal aging and decreased to 5.25 kN. No significant change was observed in the energy absorption value. A value of 66.99 joules was calculated. This value is 0.92 joules more than the value of the 500 h aged graphene-added sample.

The specific energy absorption value reached a value of 2.65 j/g with a very small increase as in EA. These results led us to think that the graphene-added glass fiber became a little more ductile with the increase in the aging time. These values obtained are higher than the values of the graphene added and unaged sample [2]. Although the increase in the aging time slightly increased the values, the EA and SEA values of the sample with no graphene added and unaged [1] could not be reached. The fiber and matrix were broken and intertwined. No flower-like opening was observed. The observations made during the experiment were in synchronization with the resulting stress–displacement curve.

Another sample, AFRPG1H2, had negative effects on hydrothermal aging values for 500 h, and decreases were observed. With the hydrothermal aging increasing from 500 h to 1000 h, EA and SEA values, except for PF value, exhibited decreases, and the values obtained were 0.9 kN, 26.84 joules and 2.32 j/g, respectively. The increase in peak force was not very big. The decreases in PF and SEA values were 31.49% and 31.56%, respectively. These values were also below the values of the unaged sample [2].

**Figure 13:**
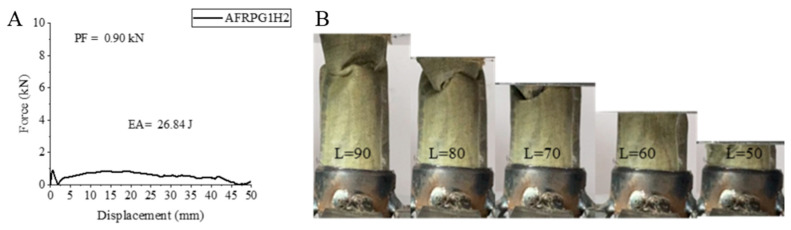
Sample AFRPG1H2: (**A**) force–displacement curve, (**B**) length changes due to deformation in quasi-static testing.

The force–displacement curve of the AFRPG1H2 sample aged for 1000 h with 0.25% graphene addition and its final state during and after the experiment are given in Figure 13. A total of 1000 h of hydrothermal aging provided flexibility for the aramid fiber sample with 0.25% graphene addition as in the 500 h aged sample. During the experiment, an apparatus was used to hold the aramid fiber sample between the pressure jaws of the device so that it would be permanent. The aramid fiber was crushed without any opening during the experiment and tended to return to its original state after the force was removed. These outcomes could also be observed in the stress–displacement curve that emerged during the experiment.

**Figure 14:**
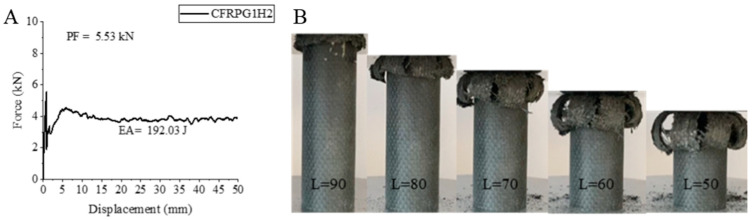
Sample CFRPG1H2: (**A**) force–displacement curve, (**B**) length changes due to deformation in quasi-static testing.

Although the CFRPG1H2 sample, which is a carbon fiber sample with 0.25% graphene addition, seems to have exhibited a slight improvement in the SEA and EA values with 1000 h of aging, these values are still below the values of the unaged sample. A 6.58% decrease in peak force compared to the CFRPG1H1 sample and a value of 5.53 kN were obtained. Increases of 120.47% and 112.14% were observed in EA and SEA values, respectively. The calculated values were 192.03 joules for EA and 7.51 j/g for SEA. While the increase in the hydrothermal aging time caused a decrease in peak force, the increase in EA and SEA values showed that water absorption gave flexibility to this sample and thus affected the area under the curve. During the test, the sample opened like a flower from inside out (Figure 14). Delamination and breakage of the matrix and fibers also occurred during the test.

**Figure 15:**
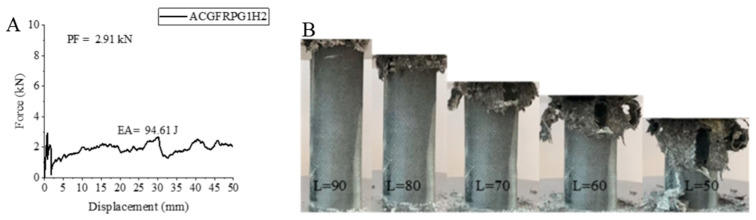
Sample ACGFRPG1H2: (**A**) force–displacement curve, (**B**) length changes due to deformation in quasi-static testing.

The situation is similar to the carbon fiber sample for the ACGFRPG1H2 sample, which is one of the hybrid samples. In this sample, the peak force value decreased while the energy absorption value and specific energy absorption value increased. The values obtained were 2.91 kN for the peak force, 94.61 joules for the energy absorption value and 3.97 j/g for the specific energy absorption. When these values are compared to the ACGFRPG1H1 sample, there is a 33.40% decrease, 71.05% increase and 60.72% increase, respectively. In Figure 15, the force–displacement curve of the ACGFRPG1H2 sample aged for 1000 h with 0.25% graphene addition and its final state during and after the experiment are given. It has been reported that the sample inside layer affects the general characteristics in these type of cylinders during the hybridization process [1]. In this sample, the aramid fiber inside layer reacted singly with decreases in all values at 1000 h of hydrothermal aging. However, the increase in EA and SEA values in this sample is evidence that other fibers show their effect in hybridization. During the experiment, it was observed that the matrix and fibers broke together with the delamination.

**Figure 16:**
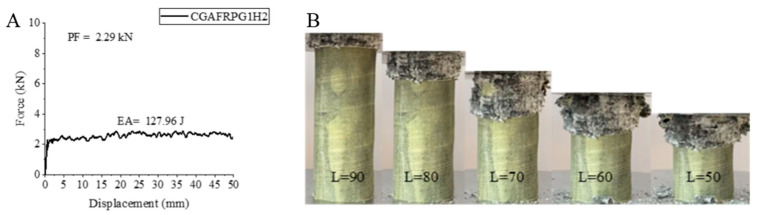
Sample CGAFRPG1H2: (**A**) force–displacement curve, (**B**) length changes due to deformation in quasi-static testing.

The other hybrid sample, CGAFRPG1H2 sample, exhibited a decrease in peak force as in other samples during the hydrothermal aging process from 500 h to 1000 h. Although the energy absorption value showed almost no change, the specific energy absorption increased. The peak force obtained in this sample was 2.29 kN, EA was 127.96 joules and SEA was 7.05 j/g. When these values are compared with the CGAFRPG1H1 sample, the peak force decreased by 28.88%, EA decreased by 0.01 joules and SEA increased by 6.17%. The obtained values are below the crush box values with 0.25% graphene addition that was not aged at all [2]. The force–displacement curve of this sample and its final state during and after the experiment are given in Figure 16. The fracture of the matrix and fibers that occurred during and after the experiment is clearly seen in Figure 15. It is also evident in its flower-like opening.

**Figure 17:**
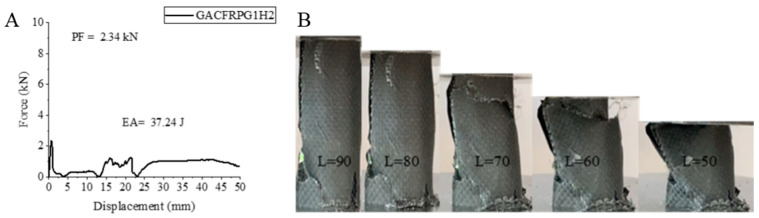
Sample GACFRPG1H2 (**A**) force–displacement curve, (**B**) length changes due to deformation in quasi-static testing.

**Figure 18:**
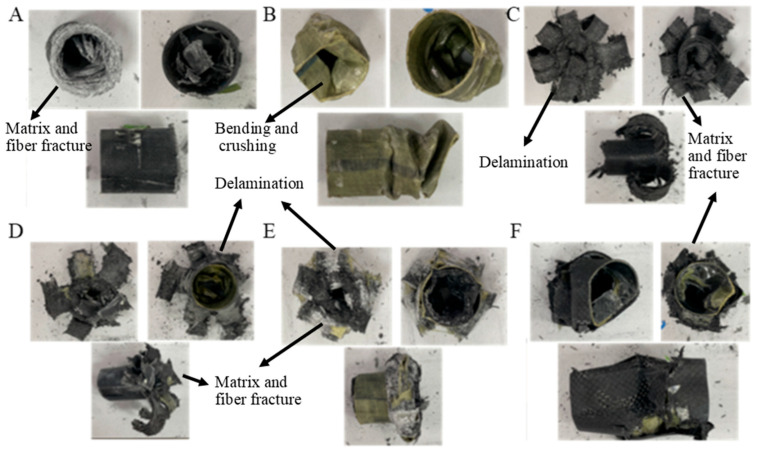
Images of samples at the end of the experiment: (**A**) GFRPG1H2, (**B**) AFRPG1H2, (**C**) CFRPG1H2, (**D**) ACGFRPG1H2, (**E**) GAFRPG1H2, (**F**) GACFRPG1H2.

The CGAFRPG1H2 sample, where the glass fiber is in the innermost part, unlike the other samples, increased the aging process, which led to an increase in all values. Naturally, this increase did not exceed the values of the 0.25 graphene-added sample that was not aged at all [2]. The peak force value obtained was 2.34 kN. This value was 20.61% higher compared to the CGAFRPG1H1 sample. EA and SEA values increased by 67.87% and 67.85%, reaching 37.24 joules and 1.88 j/g. It is clearly seen in Figure 17 that the fibers broke during the experiment. Delamination and separation are also clearly seen in Figure 18.

The values for GFRPG1H2-GACFRPG1H2 samples are given in Table 5. Images of the samples at the end of the experiment are given in Figure 18.

### 3.3. Results for GFRPG2H1—GACFRPG2H1 Samples

**Figure 19:**
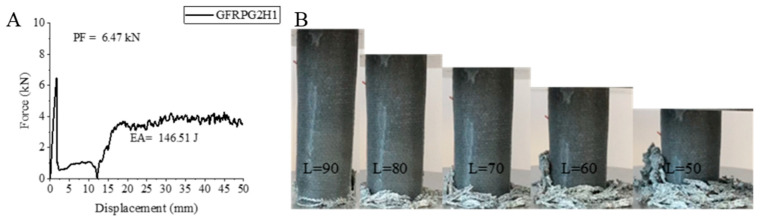
Sample GFRPG2H1: (**A**) force–displacement curve, (**B**) length changes due to deformation in quasi-static testing.

Increasing the graphene ratio to 0.50% caused significant changes in the values of the samples [2]. The 0.50% graphene-added glass fiber GFRPG2H1 sample, which was hydrothermally aged for 500 h, reacted to the aging process just like the 0.25% graphene-added GFRPG1H1 sample. All values increased compared to the unaged sample with only graphene [2]. The peak force increased by 2.37% with a value of 6.47 kN. The energy absorption value increased by 62.69% and reached 146.51 joules. The specific energy absorption also showed a similar increase with a 57.18% increase and became 5.36 j/g. If these values are compared with GFRPG1H1, it will be seen that PF, EA and SEA are 5.27% lower, 121.75% higher and 108.56% higher, respectively. These values also show the effects of increasing graphene addition on the glass fiber. The shape–displacement curve of the GFRPG2H1 sample, the length change in the sample during the experiment and the final images of the sample are given in Figure 19. In this sample, a flower-like structure was not formed; on the other hand, the fiber and matrix broke and intertwined, forming the final form.

**Figure 20:**
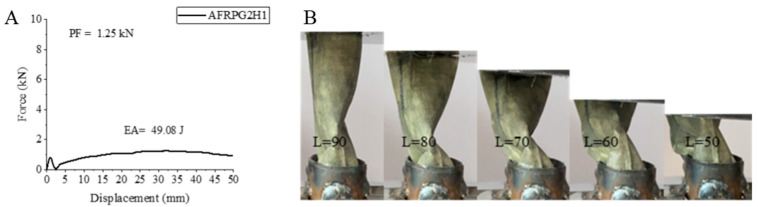
Sample AFRPG2H1: (**A**) force–displacement curve, (**B**) length changes due to deformation in quasi-static testing.

AFRPG2H1 sample, namely 0.50% graphene-added aramid fiber sample aged for 500 h, showed decreases in all values compared to the unaged 0.50% graphene-added sample [2]. The peak force value obtained was 1.25 kN. This value is 43.97% lower than the unaged 0.50% graphene-added sample [2]. EA and SEA values were calculated as 49.08 joules and 4.28 j/g. These values are 5.74% and 12.65% lower. If we compare this sample with AFRPG1H1 sample in a similar way, it will be seen that PF, EA and SEA values are 2.59%, 25.26% and 26.25% higher, respectively. As in the glass fiber sample, the increase in graphene addition had a positive effect on the sample. The strain–displacement curve and pictures of the AFRPG2H1 sample are given in Figure 20. No delamination was observed during the test. No fracture was observed and the sample only tended to bend. Although it tended to return to its original shape after the test, it could not return to its original shape.

**Figure 21:**
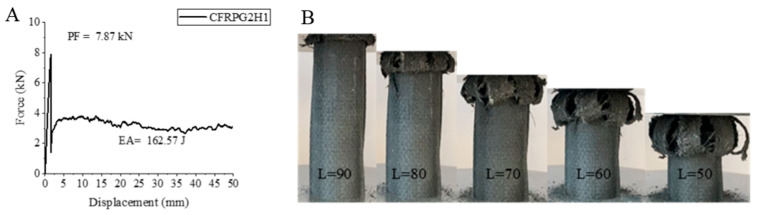
Sample CFRPG2H1: (**A**) force–displacement curve, (**B**) length changes due to deformation in quasi-static testing.

The CFRPG2H1 sample, namely carbon fiber, responded to the aging process with a decrease in its values compared to the unaged 0.50% graphene-added sample [2], just like the other samples. The peak force energy absorption value and specific energy absorption values showed decreases of 7.6%, 24.5% and 24.80%, respectively. The values obtained were 7.87 kN, 162.57 joules and 7.58 j/g, respectively. If we compare this sample with the 500 h aged CFRPG1H1 sample with 0.25% graphene added, we can observe that there is an increase of approximately 32.93% for PF, 86.64% for EA and 114.12% for SEA in PF, EA and SEA values. Here, it can be concluded that the increase in graphene addition reduces the negative effects of aging. The strain–displacement curve of the CFRPG2H1 sample and the images of this sample are given in Figure 19. As can be seen from Figure 21, the CFRPG2H1 sample was opened from inside to the outside during the test. Matrix and fiber fractures were observed along with delamination. The curve obtained during the test and the observations during the test were in full agreement with each other.

**Figure 22:**
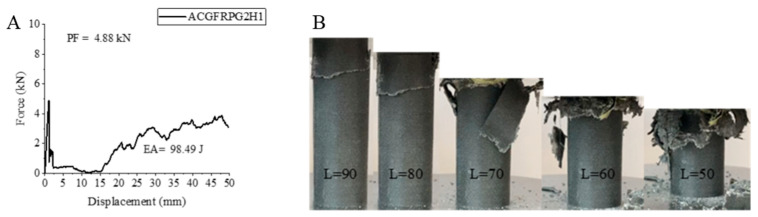
Sample ACGFRPG2H1: (**A**) force–displacement curve, (**B**) length changes due to deformation in quasi-static testing.

The first hybrid sample, ACGFRPG2H1, exhibited a decrease in its values, unlike the individual glass fiber and carbon fiber samples. It was reported that the innermost fiber is important in hybridization [1]. As its name suggests, there is aramid fiber in the innermost part of the ACGFRPG2H1 sample. The aramid fiber also showed a decrease in its values individually. The peak force showed a decrease of 32.22% with 4.88 kN compared to the unaged 0.50% graphene-added sample [2]. Energy absorption and specific energy absorption showed decreases of approximately 14.50% and 14.38%. The values obtained are 98.49 joules and 3.87 j/g. These values are higher than the ACGFRPG1H1 sample, that is, the 0.25% graphene-added sample that was hydrothermally aged for 500 h. The increases in peak force, energy absorption and specific energy absorption are 11.66%, 78.06% and 56.68%, respectively. The strain–displacement curve of the ACGFRPG2H1 sample and the pictures during and after the experiment are given in Figure 22. Fiber and matrix fractures occurred during the experiment. The broken fiber matrices opened from inside out like leaves.

**Figure 23:**
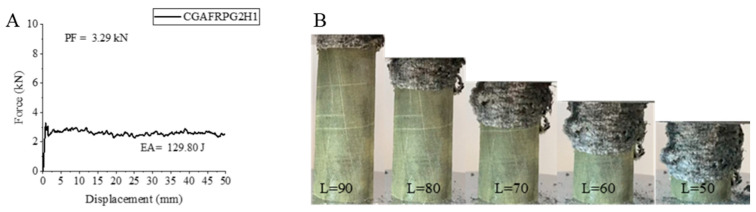
Sample CGAFRPG2H1: (**A**) force–displacement curve, (**B**) length changes due to deformation in quasi-static testing.

The CGAFRPG2H1 sample is a 500 h hydrothermal aged sample with 0.50% graphene-added and carbon fiber inner side. This sample showed an increase except for the peak force compared to the 0.50% graphene-added and unaged sample [2]. The decrease in peak force was 40.82%. The obtained value was 3.29 kN. A 60.64% increase was observed in the energy absorption value and the calculated value was 129.80 joules. This increase in energy absorption also showed itself in the specific energy absorption. An increase of 55.91% was calculated as 6.72 j/g. When we compare this sample with the CGAFRPG1H1 sample, it is seen that there were very small increases in their values. Peak force, energy absorption and specific energy absorption increased by very small rates such as 2.17%, 1.43% and 1.19%, respectively. The strain–displacement curve and pictures of the ACGFRPG2H1 sample are given in Figure 23. Breakages were observed during the experiment. Although the structure of aramid, which is caused by lower epoxy absorption compared to carbon and glass, exhibited a tendency to hold the other fibers together, it was insufficient.

**Figure 24:**
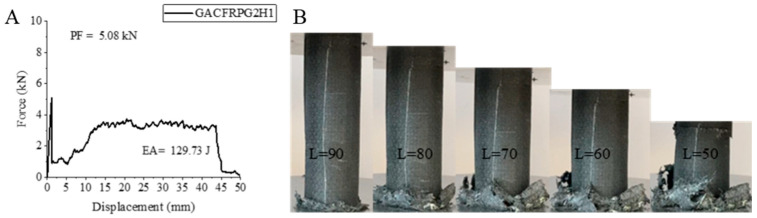
Sample GACFRPG2H1: (**A**) force–displacement curve, (**B**) length changes due to deformation in quasi-static testing.

**Figure 25:**
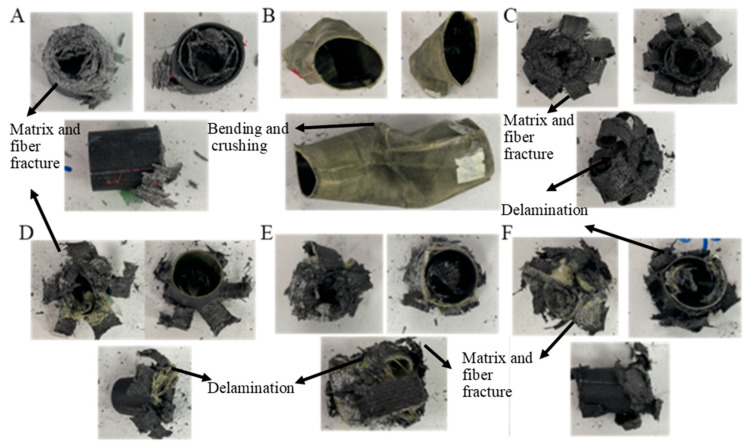
Images of samples at the end of the experiment: (**A**) GFRPG2H1, (**B**) AFRPG2H1, (**C**) CFRPG2H1, (**D**) ACGFRPG2H1, (**E**) GAFRPG2H1, (**F**) GACFRPG2H1.

The last hybrid sample, GACFRPG2H1, is a 500 h aged sample containing 0.50% graphene, in which the hybridization order was changed and the glass fiber was located in the inner part. This sample, just like the previous hybrid sample, showed a decrease in peak force compared to the unaged 0.50% graphene-added sample [2], and an increase in energy absorption and specific energy absorption. Peak force showed a decrease of 18.06% and a value of 5.08 kN. Energy absorption value showed an increase of 124.87% and a value of 129.73 joules. Specific energy absorption was also 6.43 j/g with a similar increase of 130.46%. These values were much higher than the 500 h aged 0.25% graphene-added sample, GACFRPG1H1. It is concluded that the increase in graphene addition has an effect opposite to the effect of aging. The strain–displacement curve and related images of the GACFRPG2H1 sample are given in Figure 22. As can be seen in Figure 24, during the experiment, the fiber and the matrix separated from each other and showed fractures (Figure 25).

The values for GFRPG2H1-GACFRPG2H1 samples are given in Table 6. Images of the samples at the end of the experiment are given in Figure 25.

### 3.4. Results for GFRPG2H2—GACFRPG2H2 Samples

**Figure 26:**
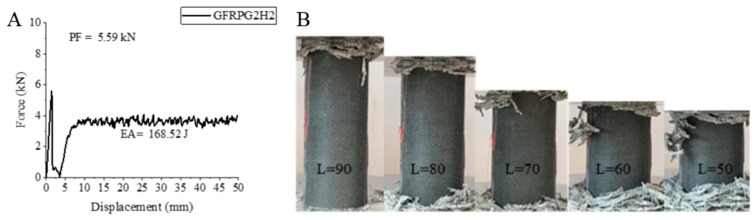
Sample GFRPG2H2: (**A**) force–displacement curve, (**B**) length changes due to deformation in quasi-static testing.

Increasing the hydrothermal aging from 500 h to 1000 h played a role in the change in the values. GFRPG2H2 sample, namely the glass fiber sample containing 0.50% graphene and aged for 1000 h, showed a decrease in peak force compared to GFRPG2H1 sample containing 0.50% graphene and aged for 500 h. However, it showed increases in energy absorption value and specific energy absorption value. This is thought to be the flexibility gained by the aging process of the glass fiber. It decreased by 13.60% in peak force and regressed to 5.59 kN. Increases of 15.02% and 19.21% were observed in energy absorption and specific energy absorption values. The values obtained were 168.52 joules and 6.39 j/g, respectively. If we compare these values of the GFRPG2H2 sample with the GFRPG1H2 sample, that is, the sample aged for 1000 h with 0.25% graphene addition, we see that there is an increase in all values. PF, EA and SEA values increased by 6.47%, 151.55% and 141.13%, respectively. This shows that the increase in the graphene addition rate has a positive effect on the glass fiber. The strain–displacement curve of the GFRPG2H2 sample and the changes in the sample during and after the test are given in Figure 26. As can be clearly seen in Figure 32, the matrix and fibers showed a tendency to break and separate from each other. These breaks were evident in the stress–displacement curve during the experiment.

**Figure 27:**
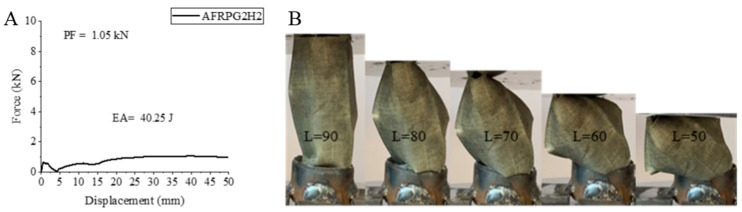
Sample AFRPG2H2: (**A**) force–displacement curve, (**B**) length changes due to deformation in quasi-static testing.

AFRPG2H2 sample, namely 1000 h hydrothermal aged aramid fiber sample with 0.50% graphene addition, responded to aging as expected. Decreases were observed in all values compared to AFRPG2H1 sample, namely, 500 h aged sample. Peak force decreased by 20.25% and became 0.63 kN. Energy absorption value decreased by 17.99% and was calculated as 40.25 joules. Specific energy absorption also decreased by 18.22% and became 3.50 j/g. AFRPG2H2 sample was aged for the same hours but increased compared to AFRPG1H2 sample with 0.25% graphene addition, except for the peak force value. PF, EA and SEA values decreased by 30%, increased by 49.96% and increased by 50.86%, respectively. It is possible to mention the positive effects of graphene addition in this sample. The shape–displacement curve of the AFRPG2H2 sample and the length changes during the experiment and the images obtained at the end of the experiment are given in Figure 27. The aramid fiber sample did not show any breakage as in the other aramid fiber samples (Figure 32). It tended to bend, and after the experiment, it showed a tendency to reach its old dimensions again, but it could not.

Although the CFRPG2H2 sample, namely the carbon fiber sample, did not seem to show much change in its values after 1000 h of hydrothermal aging, very low increases were observed. The AFRPG2H2 sample showed a 9.78% increase in peak force compared to the CFRPG2H1 sample, which was the sample with the same amount of graphene added but aged for 500 h hydrothermally, and became 8.64 kN. The energy absorption value increased by 1.94% and was calculated as 165.73 joules.

**Figure 28:**
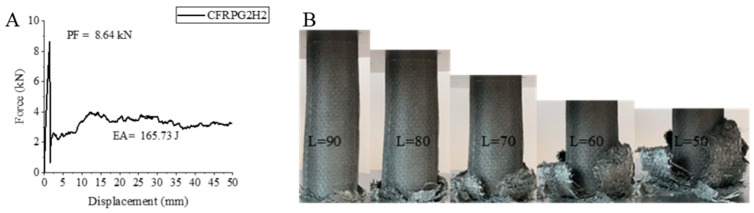
Sample CFRPG2H2: (**A**) force–displacement curve, (**B**) length changes due to deformation in quasi-static testing.

The specific energy absorption also increased by 2.63% and became 7.78 j/g. Again, if we compare this sample to the CFRPG1H2 sample with 0.25% graphene added and aged for 1000 h hydrothermally, we will observe that 56.23% increase, 13.69% decrease and 3.5% increase are encountered in PF, EA and SEA values, respectively. It has been reported that the addition of graphene does not have much effect on carbon fiber [2]. The strain–displacement curve and length change in the CFRPG2H2 sample are given in Figure 28, and the final images are given in Figure 32. During the experiment, the matrix and fibers were separated from each other and fractured, and a structure resembling a flower opening its petals was formed.

**Figure 29:**
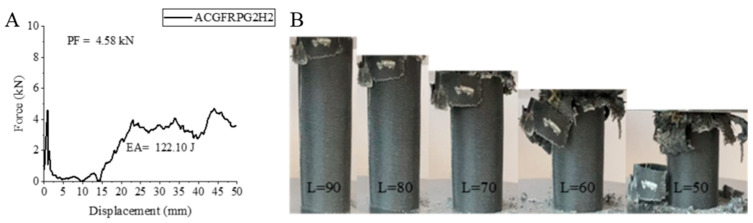
Sample ACGFRPG2H2: (**A**) force–displacement curve, (**B**) length changes due to deformation in quasi-static testing.

Hybrid sample, ACGFRPG2H2, aged for 1000 h with 0.50% graphene added responded to the extension of hydrothermal aging time with a decrease in peak force, increase in energy absorption and specific energy absorption. When compared to ACGFRPG2H1 sample, it showed a decrease of 6.14% in peak force and became 4.58 kN. Energy absorption increased by 23.97% and became 122.10 joules. Similarly, specific energy absorption increased by 24.28% and became 4.81 j/g. When compared to ACGFRPG1H2 sample aged for the same hour with 0.25% graphene-added sample, PF, EA and SEA values increased by 57.38%, 29.05% and 21.15%, respectively. The positive effects of graphene addition showed themselves. The strain–displacement curve of the ACGFRPG2H2 sample and the images obtained during and after the experiment are given in Figure 29. During the experiment, the broken pieces were shed together with delamination. The fibers tended to separate from each other (Figure 32).

The other hybrid sample, CGAFRPG2H2, is a sample containing 0.50% graphene, aged for 1000 h and in which the carbon fiber is located as a winding in the inner part. This sample showed a decrease of 2.73% in peak force compared to CGAFRPG2H1 sample containing 0.50% graphene, aged for 500 h and became 3.20 kN.

**Figure 30:**
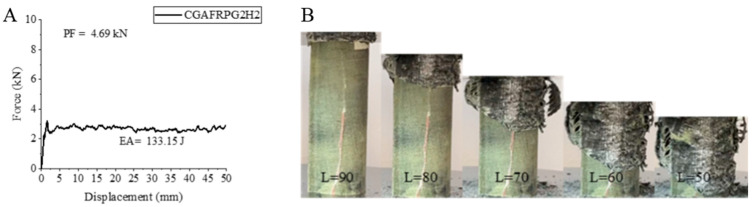
Sample CGAFRPG2H2: (**A**) force–displacement curve, (**B**) length changes due to deformation in quasi-static testing.

The energy absorption value increased by 2.58% and was calculated as 133.15 joules. The specific energy absorption value was also 6.90 j/g with a similar increase of 2.67%. When we compare the same sample with CGAFRPG1H2 sample, we can see that PF, EA and SEA values were in increase by 39.73%, increase by 4.05% and decrease by 2.12%, respectively. These values show that the increase in graphene ratio is not very effective in this sample except for the peak force. For the CGAFRPG2H2 sample, the strain–displacement curve and the pictures during and after the experiment are given in Figure 30 and Figure 32. During the experiment, separation and rupture of the matrix and fibers are observed.

Our last sample, GACFRPG2H2, is a sample with 0.50% graphene addition and 1000 h hydrothermal aged glass fiber inner side. This sample also experienced the negative effects of hydrothermal aging significantly. Compared to GACFRPG2H1 sample, with the same graphene content but 500 h hydrothermal aged sample, peak force, energy absorption and specific energy absorption values showed a decrease. Peak force, energy absorption value and specific energy absorption values were obtained as 3.76 kN, 84.26 joules and 4.02 j/g, respectively.

**Figure 31:**
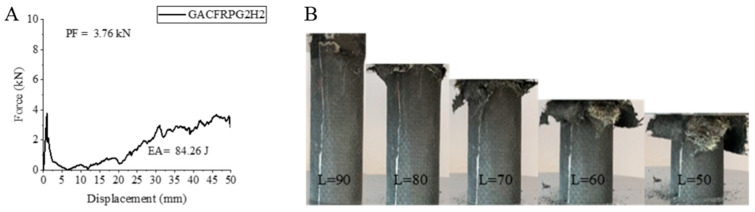
Sample GACFRPG2H2: (**A**) force–displacement curve, (**B**) length changes due to deformation in quasi-static testing.

**Figure 32:**
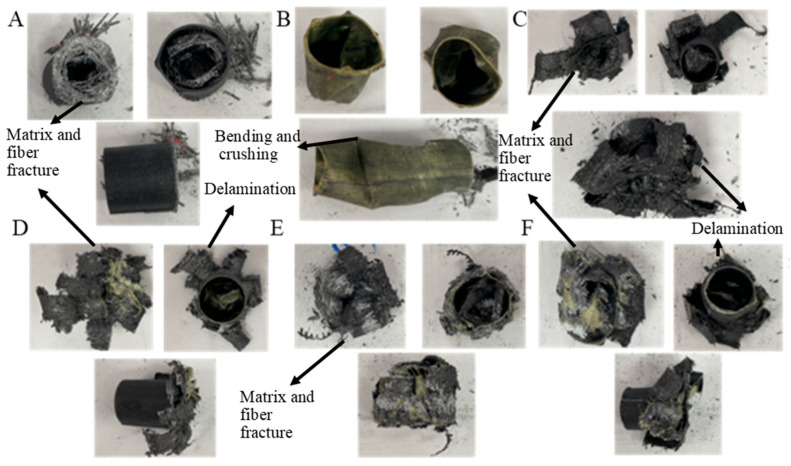
Images of samples at the end of the experiment: (**A**) GFRPG2H2, (**B**) AFRPG2H2, (**C**) CFRPG2H2, (**D**) ACGFRPG2H2, (**E**) GAFRPG2H2, (**F**) GACFRPG2H2.

The decrease rates in these values are 25.98%, 35.04% and 37.48%. Again, if we compare the same sample with GACFRPG1H2 sample with 0.25% graphene addition and aged for 1000 h, we will clearly see that the increase in PF, AE and SEA values stands out. It is obvious that the increase in graphene addition has positive effects. The increase rates are 60.68% for PF, 126.26% for EA and 113.82% for SEA. The strain–displacement curve of the GACFRPG2H2 sample, the length change during the experiment and the post-experiment pictures are given in Figure 28. As seen in Figure 31, the matrix and fibers produced fracture with delamination during the experiment. While the fibers exhibited tendency to separate from each other, they resembled the formation of a structure similar to the opening of the petals of a flower (Figure 32). All these observations are compatible with the strain–displacement curve observed during the experiment.

The values for GFRPG2H2-GACFRPG2H2 samples are given in Table 7. Images of the samples at the end of the experiment are given in Figure 25.

### 3.5. Comparison of Samples

**Figure 33:**
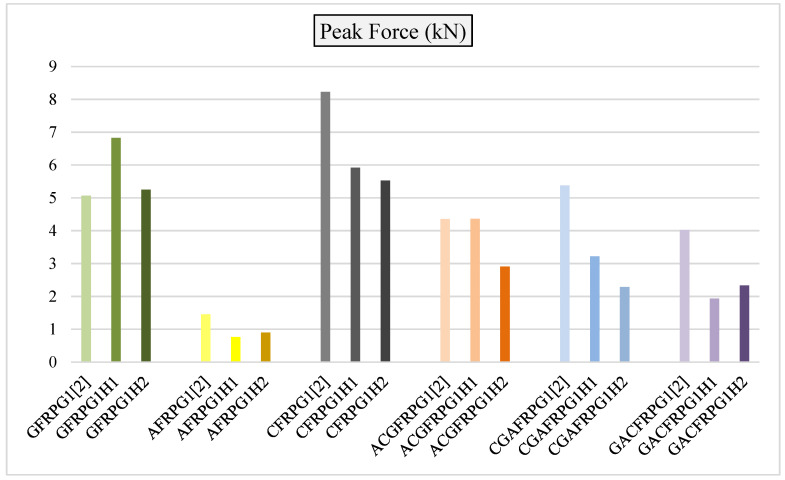
Comparison of peak forces values for samples GFRPG1H1-GACFRPG1H2.

In Figure 33, the comparative graph of the maximum peak forces of the GFRPG1H1-GACFRPG1H2 samples with the 0.25% graphene-added samples that were not hydrothermally aged, obtained in our previous study, is given.

As seen in Figure 33, hydrothermal aging generally caused negative effects on peak strengths except for the GFRPG1H1 sample. While the highest value in peak strength was in the carbon fiber sample with graphene addition that was not hydrothermally aged, the GFRPG1H1 sample had the highest value after the aging process. This obtained value is a value above the sample that was not aged at all and without graphene addition [1]. This situation is related to water absorption, which results in an increase in the flexibility of the molecular chains through internal plasticization [30]. In other samples, hydrothermal aging occasionally showed decreases or produced very small changes. While the aramid fiber sample performed a decrease with 500 h of hydrothermal aging, increasing it to 1000 h gave this sample a little more flexibility, which caused an increase in peak strength. Another similar sample is the hybrid sample, GACFRPG1H1. Carbon fiber gradually decreased in peak strength values with hydrothermal aging. The hybrid sample, CGAFRPG1H1, where the carbon fiber is located in the innermost part, also showed a similar response. The hybrid sample, ACGFRPG1H1, where the aramid fiber is located in the innermost part, showed a negligible increase in the 500 h hydrothermal aging process, but decreased in the 1000 h.

**Figure 34:**
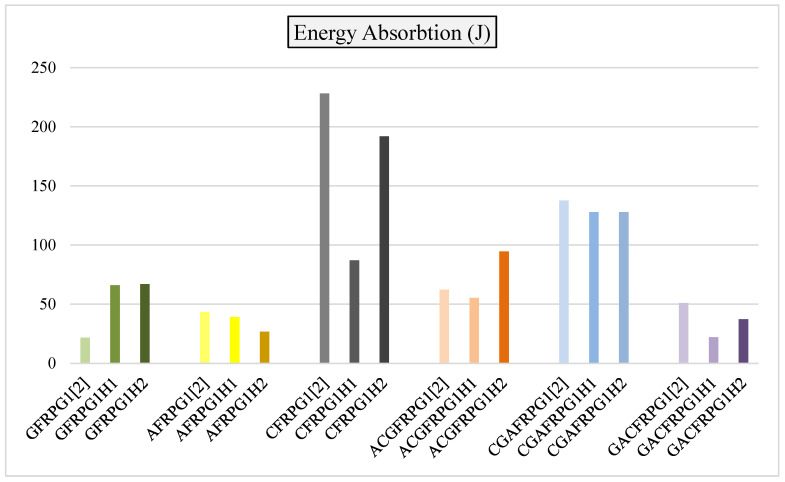
Comparison of energy absorption values for samples GFRPG1H1-GACFRPG1H2.

The comparison of energy absorption values of GFRPG1H1-GACFRPG1H2 samples is given in Figure 34. This value is also compared with the samples we obtained before, and which were not subjected to hydrothermal aging with graphene added.

As seen in Figure 34, the energy absorption values of the samples showed decreases and increases in the hydrothermal aging process. These decreases are due to the chemical changing and softening effect of the absorbed water such as plasticization and hydrolysis of the epoxy and the interface separation in graphene/epoxy and fiber/epoxy due to thermal and hygroscopic stress [31]. The glass fiber, namely the GFRPG1H1 sample, showed a continuous increase in the energy absorption value with hydrothermal aging. Similarly, in the hybrid sample where the aramid fiber was located in the inner part, the ACGFRPG1H1 sample showed a very small decrease with 500 h hydrothermal aging process, but it increased with the hydrothermal aging up to 1000 h. It is believed that this significantly unchanged behavior is due to the simultaneous activation of both hardening and embrittlement mechanisms that balance each other [32]. There was a decrease in the values of the other samples.

**Figure 35:**
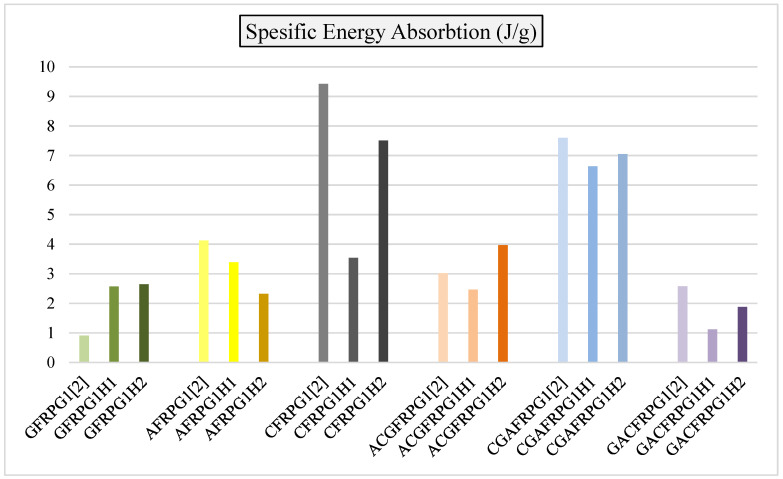
Comparison of specific energy values for samples GFRPG1H1-GACFRPG1H2.

In Figure 35, specific energy absorption values are given in comparison with the GFRPG1H1-GACFRPG1H2 samples and other samples obtained from our previous study with only 0.25% graphene addition.

As seen in Figure 35, the specific energy absorption values showed decreases and increases parallel to the energy absorption values. Only in the CGAFRPG1H2 sample, which is a hybrid sample and has carbon fiber inside, the increase in energy absorption is slightly higher than that of CGAFRPG1H1 sample, i.e., from 500 h of hydrothermal aging to 1000 h of hydrothermal aging. Since the specific energy absorption is obtained by dividing the energy absorption value by the weight of the sample, the water absorption rates affected these values, although not much. It is known that graphene addition and increasing the addition ratio affect the mechanical properties of composites [2,33,34,35,36]. In addition, hydrothermal aging is another factor affecting the mechanical properties [5,30,31,32].

**Figure 36:**
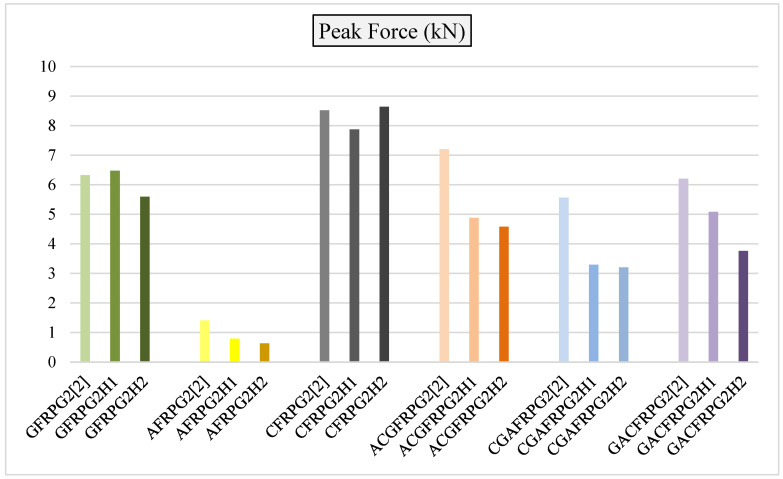
Comparison of peak forces values for samples GFRPG2H1-GACFRPG2H2.

**Figure 37:**
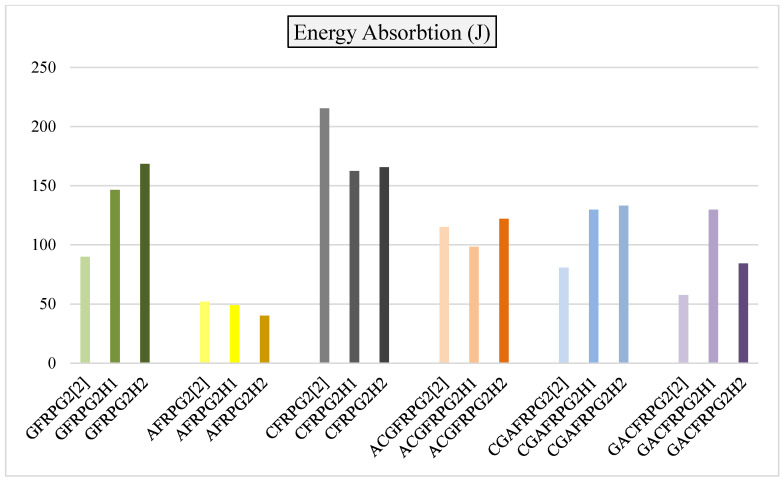
Comparison of energy absorption values for samples GFRPG2H1-GACFRPG2H2.

**Figure 38:**
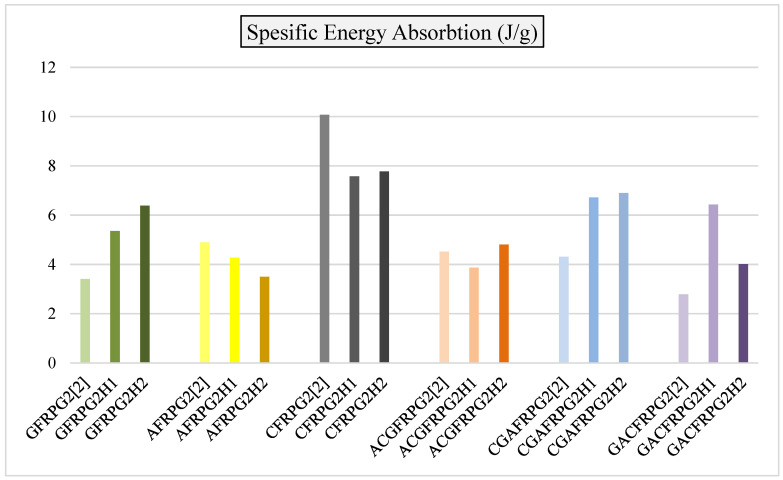
Comparison of specific energy values for samples GFRPG2H1-GACFRPG2H2.

Figure 36, Figure 37 and Figure 38 show the graphs of 500 h hydrothermal aged and 1000 h hydrothermal aged samples with 0.50% graphene addition and the unaged samples with 0.50% graphene addition obtained in our previous study. These graphs are the comparison of peak force values, energy absorption values and specific energy absorptions, respectively.

Although peak force values generally decreased in hydrothermal aging of samples with 0.50% graphene addition, very small increases were observed in it. Glass fiber, namely GFRPG2H1, showed an increase of 0.15 kN in 500 h hydrothermal aging process. Another increase was seen in CFRPG2H2 sample, but this value was a 0.12 kN increase compared to the sample not subjected to thermal aging and was a small value. Other samples showed a large decrease in peak force values with the increase in aging process time, then a relatively smaller decrease when the time was doubled. This situation is due to the fact that water absorption rate is considered to be increasing equilibrium and decreasing stage [30] and water absorption decreases or even stops after a certain period. Another reason for the decreases is that water molecules have an erosive effect on the polymer chain and eventually lead to irreversible resin hydrolysis and dissolution of interface bonds [37].

Energy absorption values increased in some samples with increasing graphene content, despite increased aging times (Figure 37). The ductile–brittle transformation of the composites due to hydrothermal aging [38] affected its mechanical properties. Increases were observed in other samples. Another important factor in these samples will be to compare the values of the hydrothermal aging process without changing according to the addition of graphene. Increasing the graphene ratio from 0.25% to 0.50% for the same hydrothermal aging period caused an increase in the values except for the CFRPG2H2 sample. The CFRPG2H2 sample showed a decrease of 26.3 joules compared to the CFRPG1H2 sample. These increases in the other samples indicate that the effects of the hydrothermal aging process decreased with the increase in graphene addition. Contrary to the expected negative effects of the aging process, the negative effects of water absorption on mechanical properties in artificial hydrothermal aging were reduced in some samples with the presence of graphene [39].

Specific energy absorption values also showed decreases and increases parallel to energy absorption values. While GFRPG2H1 sample and hybrid samples ACGFRPG2H2, CGAFRPG2H2 and GACFRPG2H2 samples reacted positively to hydrothermal aging process, there were decreases in carbon fiber, namely CFRPG2H1 and AFRPG2H1 samples (Figure 38). Aging and moisture can cause decreases in crushing properties [40,41]. Again, if we compare these values with 0.25% graphene-added and 500 and 1000 h hydrothermal aged samples, we see the effects of graphene addition in general. In other words, all values increased compared to 0.25% graphene-added hydrothermal aged samples. Only in CGAFRPG2H2 sample, which is the hybrid sample with carbon fiber inside, an insignificant decrease of 0.15 j/g was observed compared to CGAFRPG1H2 sample.

It is known that the good interfacial connection between the fiber and matrix in composite materials affects their damage behavior. In addition, while the added nanoparticles sometimes strengthen these interfacial connections, when added in excess, the partially larger areas formed by the aggregation of nanoparticles can trigger the initiation of damage and lead to earlier failure [29]. Aging also has negative effects on the materials. Water absorption is also a known fact that affects the mechanical properties of the material [8,9,10]. Sometimes, the use of nanoparticles reduces water absorption, which can mitigate the negative effects on the material.

Since the lightness of composite materials is a key feature for vehicles, the data obtained in such studies highlight the importance of using composite crash boxes in vehicles. The data obtained from experimental results offer pioneering values for practical applications.

## 4. Conclusions

In this study, epoxy resin matrix glass, aramid and carbon fiber composite crush boxes produced with 0.25% and 0.50% graphene addition were subjected to 500 h and 1000 h of hydrothermal aging. After hydrothermal aging process, peak forces, energy absorptions and specific energy absorptions of the crush boxes were investigated experimentally. The effects of hydrothermal aging on fiber structure, hybridization of fibers and graphene addition ratio depending on time were investigated experimentally. In general, the results are listed below:

➢ The highest peak force value was observed in the CFRPG2H2 sample, which is a sample containing 0.50% graphene subjected to 1000 h of hydrothermal aging. The highest specific energy absorption value is also in this sample.
➢ Among the samples the highest energy absorption value was obtained with the CFRPG1H2 sample, which is another carbon sample, that is, the sample that was hydrothermally aged for 500 h and contained 0.50% graphene.
➢ The lowest peak force was obtained in the AFRPG2H2 sample with aramid fiber, which has low epoxy absorption.
➢ The lowest energy absorption value and specific energy absorption value were observed in the hybrid sample GACFRPG1H1, which contained 500 h aged glass fiber with 0.25% graphene addition inside.
➢ Although decreases were generally observed in the first stage of the hydrothermal aging process, increases were observed in some samples with the extension of the aging period.
➢ As the graphene addition rate increased, general increases were observed in the values of the samples. These increases revealed that the increase in graphene addition reduced the effects of hydrothermal aging.
➢ As a result, carbon fiber-reinforced crush boxes are recommended as crush boxes.

## Figures and Tables

**Table 1:** Specific properties of fibers used for production.

Fibers	Density (g/cm^3^)	Tensile Strength (MPa)	Poisson’s Ratio	Elongation (%)	Young’s Modulus (GPa)	Shear Modulus (GPa)
Glass	1.3	3400	0.22	1.8–3.2	76	33
Carbon	1.5	4300–4600	0.38	1.75–1.98	2550	110
Aramid	1.4	3000–3150	0.279	2.5–3.6	67	16.2

**Table 2:** Properties of epoxy resin used in production.

Density (g/cm^3^)	1.13–1.17
Viscosity (mPas)	700–900
Operating temperature before applying heat treatment (°C)	−60 to +50
Operating temperature after applying heat treatment (°C)	−60 to +80
Application temperature (°C)	+10 to +50

**Table 3:** Names and specific characteristics of samples.

Samples	Fiber Type *	Graphene Ratio (%)	Hydrothermal Aging Time (Hours)
GFRPG1H1	Glass	0.25	500
AFRPG1H1	Aramid	0.25	500
CFRPG1H1	Carbon	0.25	500
ACGFRPG1H1	Aramid–Carbon–Glass	0.25	500
CGAFRPG1H1	Carbon–Glass–Aramid	0.25	500
GACFRPG1H1	Glass–Aramid–Carbon	0.25	500
GFRPG1H2	Glass	0.25	1000
AFRPG1H2	Aramid	0.25	1000
CFRPG1H2	Carbon	0.25	1000
ACGFRPG1H2	Aramid–Carbon–Glass	0.25	1000
CGAFRPG1H2	Carbon–Glass–Aramid	0.25	1000
GACFRPG1H2	Glass–Aramid–Carbon	0.25	1000
GFRPG2H1	Glass	0.50	500
AFRPG2H1	Aramid	0.50	500
CFRPG2H1	Carbon	0.50	500
ACGFRPG2H1	Aramid–Carbon–Glass	0.50	500
CGAFRPG2H1	Carbon–Glass–Aramid	0.50	500
GACFRPG2H1	Glass–Aramid–Carbon	0.50	500
GFRPG2H2	Glass	0.50	1000
AFRPG2H2	Aramid	0.50	1000
CFRPG2H2	Carbon	0.50	1000
ACGFRPG2H2	Aramid–Carbon–Glass	0.50	1000
CGAFRPG2H2	Carbon–Glass–Aramid	0.50	1000
GACFRPG2H2	Glass–Aramid–Carbon	0.50	1000

* Single or fiber winding sequence from inside to outside.

**Table 4:** Data obtained and calculated from the quasi-static test results of GFRPG1H1-GACFRPG1H1 composite crush boxes.

Tubes	F Maximum (kN)	Energy Absorption (J)	Weight (g)	Specific Energy Absorption J/g
GFRPG1H1	6.83	66.07	25.65	2.57
AFRPG1H1	0.77	39.18	11.54	3.39
CFRPG1H1	5.92	87.10	24.59	3.54
ACGFRPG1H1	4.37	55.31	22.31	2.47
CGAFRPG1H1	3.22	127.97	19.27	6.64
GACFRPG1H1	1.94	22.18	19.77	1.12

**Table 5:** Data obtained and calculated from the quasi-static test results of GFRPG1H2-GACFRPG1H2 composite crush boxes.

Tubes	F Maximum (kN)	Energy Absorption (J)	Weight (g)	Specific Energy Absorption (J/g)
GFRPG1H2	5.25	66.99	25.25	2.65
AFRPG1H2	0.90	26.84	11.56	2.32
CFRPG1H2	5.53	192.03	25.56	7.51
ACGFRPG1H2	2.91	94.61	23.83	3.97
CGAFRPG1H2	2.29	127.96	18.14	7.05
GACFRPG1H2	2.34	37.24	19.77	1.88

**Table 6:** Data obtained and calculated from the quasi-static test results of GFRPG2H1-GACFRPG2H1 composite crush boxes.

Tubes	F Maximum (kN)	Energy Absorption (J)	Weight (g)	Specific Energy Absorption (J/g)
GFRPG2H1	6.47	146.51	27.33	5.36
AFRPG2H1	0.79	49.08	11.46	4.28
CFRPG2H1	7.87	162.57	21.43	7.58
ACGFRPG2H1	4.88	98.49	25.41	3.87
CGAFRPG2H1	3.29	129.80	19.29	6.72
GACFRPG2H1	5.08	129.73	20.17	6.43

**Table 7:** Data obtained and calculated from the quasi-static test results of GFRPG2H2-GACFRPG2H2 composite crush boxes.

Tubes	F Maximum (kN)	Energy Absorption (J)	Weight (g)	Specific Energy Absorption (J/g)
GFRPG2H2	5.59	168.52	26.35	6.39
AFRPG2H2	0.63	40.25	11.47	3.50
CFRPG2H2	8.64	165.73	21.30	7.78
ACGFRPG2H2	4.58	122.10	25.35	4.81
CGAFRPG2H2	3.20	133.15	19.29	6.90
GACFRPG2H2	3.76	84.26	20.95	4.02

## Data Availability

The raw data supporting the conclusions of this article will be made available by the authors on request.

## References

[1] Erkek B., Kosedag E., Adin H. (2024). Hybridization effect on energy absorption capacity of composite crash boxes. Polym. Compos..

[2] Erkek B., Kosedag E., Adin H. (2024). The impact of graphene filler on the energy absorption of hybrid composite crash boxes. Int. J. Mech. Mater. Des..

[3] Bockenheimer C., Fata D., Possart W. (2004). New aspects of aging in epoxy networks. II. Hydrothermal aging. J. Appl. Polym. Sci..

[4] El-baky M.A., Attia M. (2019). Water absorption effect on the in-plane shear properties of jute–glass–carbon-reinforced composites using Iosipescu test. J. Compos. Mater..

[5] Chakraverty A.P., Mohanty U.K., Mishra S.C., Biswal B.B. (2017). Effect of Hydrothermal immersion and Hygrothermal Conditioning on Mechanical Properties of GRE Composite. IOP Conf. Ser. Mater. Sci. Eng..

[6] Soykok I.F., Sayman O., Ozen M., Korkmaz B. (2013). Failure analysis of mechanically fastened glass fiber/epoxy composite joints under thermal effects. Compos. Part B Eng..

[7] Mourad A.-H.I., Abdel-Magid B.M., El-Maaddawy T., Grami M.E. (2010). Effect of Seawater and Warm Environment on Glass/Epoxy and Glass/Polyurethane Composites. Appl. Compos. Mater..

[8] Malik M., Saxena P. (2025). A comparative assessment of hydrothermal aging effects on performance and failure modes of additively manufactured continuous basalt fiber-reinforced thermoplastic composites. Compos. Part Appl. Sci. Manuf..

[9] Zhang X., Xu X., Yang Y., Hao J., Wu Z. (2025). Hydrothermal aging of carbon fiber reinforced polyether ether ketone composites: Behavior, mechanism and life prediction. Polym. Compos..

[10] Glaskova-Kuzmina T., Pinto R., Zukiene K., Monastyreckis G., Spacek V., Zeleniakiene D. (2025). Hydrothermal Aging of Basalt Fiber-Reinforced Epoxy Composites Modified with Star-Like Polymer. Polym. Compos..

[11] Yu P., Yu Z., Zhou X., Xu W. (2023). Crash Performance of Inward-Inverting Composite Tubes Filled with Foam: Experimentation and Simulation. Materials.

[12] Dirgantara T., Jusuf A., Kurniati E.O., Gunawan L., Putra I.S. (2018). Crashworthiness analysis of foam–filled square column considering strain rate effect of the foam. Thin-Walled Struct..

[13] Djamaluddin F. (2024). Optimization of foam-filled crash-box under axial loading for pure electric vehicle. Results Mater..

[14] Kosedag E. (2024). The effect of halloysite nanotubes reinforced epoxy filler on the crushing behavior of aluminum tubes. J. Appl. Polym. Sci..

[15] Kösedağ E. (2023). Investigation of the Effect of Filling Ratio on Mechanical Properties of Pumice Filled Epoxy-Based Composites. Gazi Üniv. Fen Bilim. Derg. Part C Tasar. Teknol..

[16] De Biasio A., Ghasemnejad H., Srimanosaowapak S., Watson J.W. (2025). Development of multi aluminium foam-filled crash box systems to improve crashworthiness performance of road Service vehicle. Eur. J. Mech.-A Solids.

[17] Nguyen H., Nguyen D., Tran H., Tran-van T. (2025). Investigation on multi-corners crash-box structure subjected to axial crushing. J. Phys. Conf. Ser..

[18] Ciampaglia A., Patruno L., Ciardiello R. (2024). Design of a Lightweight Origami Composite Crash Box: Experimental and Numerical Study on the Absorbed Energy in Frontal Impacts. J. Compos. Sci..

[19] El-baky M.A.A., Allah M.M.A., Kamel M., Abdel-Aziem W. (2023). Fabrication of Glass/Jute Hybrid Composite over Wrapped Aluminum Cylinders: An Advanced Material for Automotive Applications. Fibers Polym..

[20] Qin X., Ma Q., Gan X., Cai M., Cai W. (2023). Failure analysis and multi-objective optimization of crashworthiness of variable thickness Al-CFRP hybrid tubes under multiple loading conditions. Thin-Walled Struct..

[21] Zhu G., Zhao X., Shi P., Yu Q. (2019). Crashworthiness Analysis and Design of Metal/CFRP Hybrid Structures Under Lateral Loading. IEEE Access.

[22] Zhu G., Sun G., Liu Q., Li G., Li Q. (2017). On crushing characteristics of different configurations of metal-composites hybrid tubes. Compos. Struct..

[23] Zhang X., Zhang H., Wen Z. (2015). Axial crushing of tapered circular tubes with graded thickness. Int. J. Mech. Sci..

[24] Yildirim H., Pihtili H., Çetïnkaya Ş. (2016). Investigation of torsional behaviors of carbon/epoxy shafts at a different orientation angles by experimental and finite element method which manufactured with filament winding method using composite materials. Eur. J. Technol..

[25] Kim J.-S., Yoon H.-J., Shin K.-B. (2011). A study on crushing behaviors of composite circular tubes with different reinforcing fibers. Int. J. Impact Eng..

[26] El-baky M.A.A., Allah M.M.A., Kamel M., Abd-Elaziem W. (2022). Lightweight cost-effective hybrid materials for energy absorption applications. Sci. Rep..

[27] Alshahrani H., Sebaey T.A., Hegazy D.A., El-Baky M.A.A. (2022). Effects of halloysite clay nanotubes on the energy absorption and failure mechanisms of glass/epoxy composite tubes subjected to quasi-static axial crushing. Polym. Compos..

[28] Alshahrani H., Sebaey T.A., Hegazy D.A., El-baky M.A.A. (2022). Development of efficient energy absorption components for crashworthiness applications: An experimental study. Polym. Adv. Technol..

[29] Gupta R., Kumar G., Bisaria H., Zafar S. (2025). Effect of graphene nanoparticles on electrical, mechanical and viscoelastic behavior of CFRP multifunctional multiscale composites. Polym. Compos..

[30] Li R., Ye L., Li G. (2018). Long-Term Hydrothermal Aging Behavior and Aging Mechanism of Glass Fibre Reinforced Polyamide 6 Composites. J. Macromol. Sci. Part B.

[31] Fulmali A.O., Sen B., Nayak B.A., Prusty R.K. (2021). Effect of repeated hydrothermal cycling on the durability of glass fiber/epoxy composites with and without carbon nanotube reinforcement. Polym. Compos..

[32] Alessi S., Pitarresi G., Spadaro G. (2014). Effect of hydrothermal ageing on the thermal and delamination fracture behaviour of CFRP composites. Compos. Part B Eng..

[33] Ahmad M.A., Ridzuan M.J., Majid M.A., Sapuan S.M., Ismail M.S., Razlan Z.M., Shahriman A.B. (2024). Thermal behaviour of graphene nanoplatelets and multiwalled carbon nanotubes filled-glass fibre-reinforced epoxy composites. J. Therm. Anal. Calorim..

[34] Bedi S.S., Mallesha V., Mahesh V., Mahesh V., Ponnusami S.A. (2024). Investigation of low-percentage graphene reinforcement on the mechanical behaviour of additively manufactured polyethylene terephthalate glycol composites. J. Thermoplast. Compos. Mater..

[35] Chen D., Li J., Sun K., Fan J. (2023). Graphene-reinforced metal matrix composites: Fabrication, properties, and challenges. Int. J. Adv. Manuf. Technol..

[36] El-Ghazaly A., Anis G., Salem H.G. (2017). Effect of graphene addition on the mechanical and tribological behavior of nanostructured AA2124 self-lubricating metal matrix composite. Compos. Part Appl. Sci. Manuf..

[37] Xian G., Bai Y., Qi X., Wang J., Tian J., Xiao H. (2024). Hygrothermal aging on the mechanical property and degradation mechanism of carbon fiber reinforced epoxy composites modified by nylon 6. J. Mater. Res. Technol..

[38] Fang M., Zhang N., Huang M., Lu B., Lamnawar K., Liu C., Shen C. (2020). Effects of Hydrothermal Aging of Carbon Fiber Reinforced Polycarbonate Composites on Mechanical Performance and Sand Erosion Resistance. Polymers.

[39] Sepetcioglu H., Gunoz A., Kara M. (2021). Effect of hydrothermal ageing on the mechanical behaviour of graphene nanoplatelets reinforced basalt fibre epoxy composite pipes. Polym. Polym. Compos..

[40] Zuo W., Luo Q., Li Q., Sun G. (2023). Effect of thermal and hydrothermal aging on the crashworthiness of carbon fiber reinforced plastic composite tubes. Compos. Struct..

[41] Oğuz Z.A., Özbek Ö., Erkliğ A., Bozkurt Ö.Y. (2023). Hydrothermal aging effect on crushing characteristics of intraply hybrid composite pipes. Eng. Struct..

